# Molecular analysis and genotype-phenotype correlations in patients with classical congenital adrenal hyperplasia due to 21-hydroxylase deficiency from southern Poland — experience of a clinical center

**DOI:** 10.1007/s42000-022-00348-z

**Published:** 2022-01-26

**Authors:** Anna Kurzyńska, Anna Skalniak, Kim Franson, Viola Bistika, Alicja Hubalewska-Dydejczyk, Elwira Przybylik-Mazurek

**Affiliations:** 1 Clinical Department of Endocrinology, Jagiellonian University Medical College, Krakow, Poland; 2 Karolinska Institute, Stockholm, Sweden; 3 Faculty of Medicine, Hacettepe University, Ankara, Turkey

**Keywords:** Congenital adrenal hyperplasia, *CYP21A2*, Genotype-phenotype correlation, 21-hydroxylase deficiency

## Abstract

**Purpose:**

The prevalence of *CYP21A2* gene variants and genotype-phenotype correlations are variable among populations. The aim of this study was to characterize *CYP21A2* gene variants in adult patients with classical congenital adrenal hyperplasia (CCAH) from southern Poland and to analyze genotype-phenotype correlations.

**Materials/Methods:**

A total of 48 patients (30 women and 18 men) with CCAH were included in the study. Patients were divided into two clinical subgroups, namely, salt-wasting (SW) — 38 patients and simple virilizing (SV) — 10 patients. A genetic analysis MLPA (multiplex ligation-dependent probe amplification) was performed in all of them. In dubious cases, the analysis was complemented by Sanger sequencing. Genotypes were classified into five groups (depending on the residual in vitro enzymatic activity), namely, *null*, A, B, C, and D, and correlated with the clinical picture.

**Results:**

Molecular defects were investigated and identified in 48 patients. The most common variant in the studied group was I2G, followed by whole or partial gene copy deletion, and I172N. One novel variant c.[878G>T] (p.Gly293Val) was found. In nine patients, a non-concordance between genotype and phenotype was observed. Genotype-phenotype correlations measured by positive predictive value (PPV) were as follows: 100% in group *null*, 90.5% in group A, and 66.7% in group B.

**Conclusions:**

*CYP21A2* variants in the studied cohort were similar to values previously reported in other countries of the region. There was a good correlation between genotype and phenotype in the *null* and A groups, the correlation being considerably lower in group B.

## Introduction

Congenital adrenal hyperplasia (CAH) is an endocrine disorder caused by mutations of genes coding for the synthesis of enzymes involved in adrenal steroidogenesis. Based on the type of enzyme block, several CAH types can be distinguished. The most common type (which accounts for about 95–99% of cases) is related to mutation in the *CYP21A2* gene, encoding 21-hydroxylase, and may result in different clinical forms, namely, classical, including salt- wasting (SW) and simple virilizing (SV), and nonclassical (NCCAH), the latter manifesting at a later age and characterized by milder symptoms [1–3]. The incidence of CAH is estimated at about 1:15,000 and differs among populations [4]. In some ethnic groups, the reported prevalence is higher (for example, in the population of La Réunion, it is 1:2141 [5], and among Yupik Eskimos, it is 1:280 [6]). In the Caucasian population, the prevalence of the disease varies from 1 in 5000 to 1 in 23,000 live births [7]. According to the latest data, in the USA, the incidence of CAH varies from 1:9941 to 1:28,661 live births [8]. The *CYP21A2* gene is located on the short arm of chromosome 6 (6p21.3) [9]. The inactive pseudogene, *CYP21A1P*, is 98% homologous compared to the active form of *CYP21A2* and contains variants which, when incorporated in the active gene, can lead to the loss of its functions. The majority of pathogenic variants (about 90–95%) occurring within *CYP21A2* originate from this pseudogene. Within the active gene, there are also de novo variants causing only a small portion of inherited cases of CAH [10]. Different variants in the *CYP21A2* gene can lead to a variable degree of loss of 21-hydroxylase activity, which can result in various clinical presentations. To date, over 200 variants with a pathogenic role have been described [11, 12]. The majority of pathogenic variants in the CYP21A2 gene are large conversions, large deletions, or one of nine small variants, as follows: p.Gly111Valfs*21 (classically designated as “del8bp” or “Δ8bp”), exon 6 (“E6”) cluster (p.[Ile237Asn; Val238Glu; Met240Lys]), p.Leu308Phefs*6 (“F306+T”), p.Gln319Ter (“Q318X”), p.Arg357Trp (“R356W”), p.Ile173Asn (“I172N”), p.Pro31Leu, p.Val282Leu, p.Pro454Ser, and c.293-13A/C>G (“I2G”) [13]. Molecular defects in Polish patients with CAH have not so far been analyzed. Therefore, the aim of our study was to identify the spectrum of variants in adult patients from southern Poland with classical congenital adrenal hyperplasia (CCAH) due to 21-hydroxylase deficiency and to analyze genotype-phenotype correlations.

## Materials and methods

### Study population

Forty-eight adult patients diagnosed with CCAH due to 21-hydroxylase deficiency, treated at the Department of Endocrinology, University Hospital Medical College, Krakow, Poland, between 2015 and 2019 were enrolled in this study. In the study group, there were 30 women aged 28.20 (± 12.12) years and 18 men aged 27.70 (± 8.97) years. In total, 38 patients (79.17%) had the SW form and 10 (20.83%) the SV form. The CCAH diagnosis was made based on the clinical and physical examination and retrospective analysis of the patients’ medical history. Data such as age at onset of the disease, electrolyte disturbances, genital appearance, previous urological surgery, evidence of hyperandrogenism, hormonal data including 17-hydroxyprogesterone (17-OHP), ACTH, plasma renin activity (PRA), aldosterone levels, and treatment scheme were taken into consideration. Patients who presented symptoms of adrenal crisis as neonates were classified in the SW group. Patients diagnosed in early childhood, but without salt-wasting form symptoms, were assigned to the SV group.

This study is in compliance with the 1964 Declaration of Helsinki and its later amendments. All patients signed an informed consent for participation in the study; an additional informed consent for the genetic analysis was also obtained. The study was approved by the Ethics Committee of the Jagiellonian University Medical College: KBET/225/B/2013.

### Statistical analysis

Continuous data with normal distribution are presented as mean value and standard deviation (SD), and non-normal variables are reported as median and interquartile range (IQR) (Me [Q1;Q3]). Categorical data are presented as percentages. Due to the small size of the groups, continuous data were compared using the Kruskal-Wallis test. Categorical data were compared using the chi-square test. A *P*-value below 0.05 was considered statistically significant. To obtain a visual representation of global patterns within the data, correspondence analysis was implemented. Data were analyzed using Statistica 13.0.

### Molecular analysis

DNA was extracted from peripheral blood samples using the NucleoSpin Blood kit (Macherey-Nagel Inc.), according to the manufacturer’s protocol. Genetic analysis based on MLPA (multiplex ligation-dependent probe amplification), with the use of the probemix SALSA MLPA P050 CAH from MRC Holland, was performed according to the manufacturer’s recommendations, using 50 ng of the isolated DNA per sample. The SALSA MLPA Probemix from MRC Holland, which enables detection of large rearrangements and seven of the most common point mutations in one reaction mix at the same time, was used as the first step of molecular testing of our studied group. In dubious cases, it was complemented by Sanger sequencing, based on the method published by Xu et al. [14]. For the long-range PCR reaction, Amplus polymerase (EurX Sp. z o.o.) was used according to the manufacturer’s recommendations, with 200 ng DNA per 25 μl reaction volume and betaine (Sigma-Aldrich) added to the reaction mixture at a final concentration of 1M. The reaction was performed on an Eppendorf Mastercycler realplex thermocycler with an annealing temperature of 61°C and 20 sec added to each elongation step beginning from cycle 21. After agarose gel verification, the remaining reaction mixture was purified with NucleoSpin PCR Clean-up (Macherey-Nagel). The sequencing PCR reaction was performed with BigDye Terminator v3.1 (ThermoFisher Applied Biosystems) and 120 ng of the purified PCR product in 10 μl reaction volume. The sequencing conditions were in accordance with the manufacturer’s recommendations, with an annealing temperature of 55 °C. Products were purified by ethanol precipitation, and pellets were resuspended in 20 μl nuclease-free water for capillary electrophoresis (ABI 3500, Applied Biosystems).

**Fig. 1:**
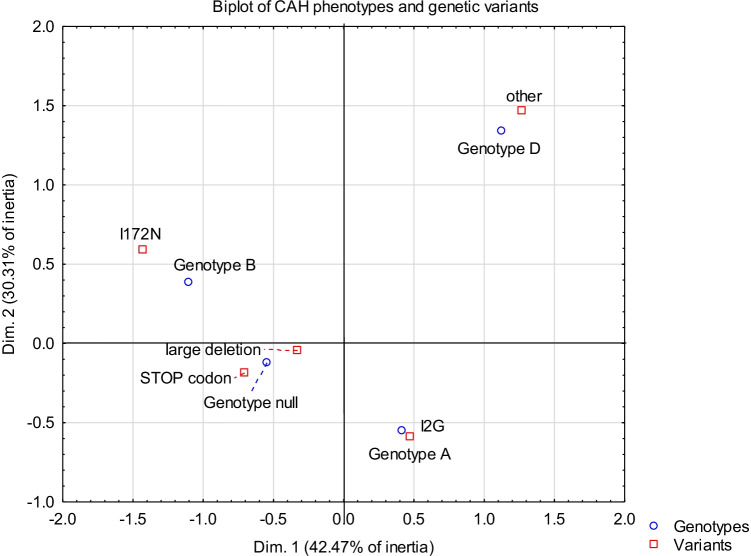
*CYP21A2* variants found in 44 patients (88 alleles) with CCAH

Four patients were excluded from further analysis because it was not possible to determine the distribution of variants on individual alleles without performing molecular analysis of the patients’ parents, who were not available for testing. Genotypes were categorized into five groups (according to the published residual in vitro activity of 21-hydroxylase, based on literature data [15, 16]), namely, groups *null*, A, B, C, and D, and then compared with the clinical presentation (excepting group C, which was excluded from further analyses). Group *null*, with 0% residual enzyme function, included patients with alterations found in both alleles of *CYP21A2*, causing a total absence of enzymatic activity (deletions, gene conversions, F306+T, del8bp, cluster E6, R356W, and Q318X). Group A (0–1% residual 21-hydroxylase activity) comprised patients who were I2G homozygotes or heterozygotes consisting of I2G and another variant, with minimal enzymatic activity (0–1%). Homozygotic patients with the variant I172N (approximately 2% of residual 21-hydroxylase enzyme activity) or heterozygotes with the *null*, A, or B group variants were assigned to group B, with 1–2% residual enzyme activity. Genotype group C (patients with moderately impaired 21-hydroxylase activity and with about 20–60% preserved residual enzymatic function) were homozygotes or heterozygotes of the milder variants. Patients who were carriers of variants of unknown in vitro impact on residual enzymatic activity were assigned to genotype group D. The global representation of these classes with different *CYP21A2* variants is depicted in Fig. 1. The presented variants are numbered according to reference sequences NM_000500.9 (at DNA level) and NP_000491.4 (at protein level). Additionally, common genetic variants are referred to in accordance with classically used designations to maintain coherence with other literature sources.

## Results

**Fig. 2:**
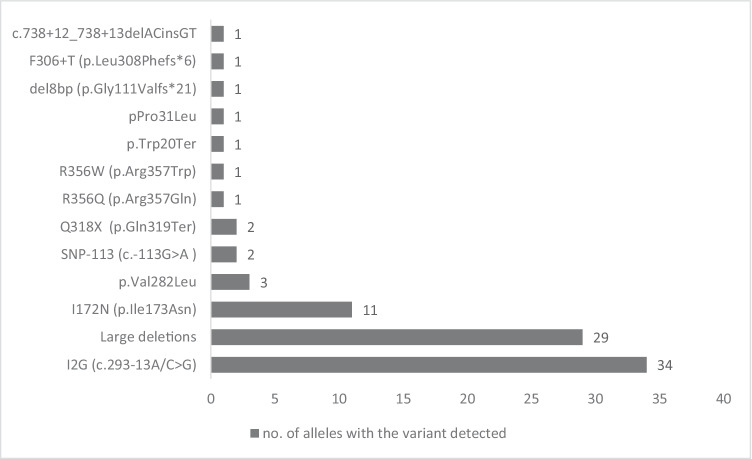
Visual representation of data profiles — CAH genotypes and genetic variants

Among the group of 44 patients with CCAH (88 alleles), *CYP21A2* gene mutations were detected in all of them*.* A total of 100% of the analyzed 88 alleles revealed a mutation of the *CYP21A2* gene. The *CYP21A2* gene variants found in our study group are shown in Fig. 2.

The most common alteration in CCAH patients was the I2G (c.293-13A/C>G) variant with an allele frequency of 38.63%, followed by large deletions and I172N (p.Ile173Asn) (32.95% and 12.5% of all alleles, respectively). Other detected variants, from the most to the least prevalent, were as follows: p.Val282Leu, SNP-113 (c.-113G>A), Q318X (p.Gln319Ter), R356Q (p.Arg357Gln), R356W (p.Arg357Trp), p.Trp20Ter, p.Pro31Leu, del8bp (p.Gly111Valfs*21), F306+T (p.Leu308Phefs*6), and c.738+12_738+13delACinsGT.

Genetic variant distribution among the 88 alleles according to genotype groups *null*, A, B, and D is presented in Table 1.

**Table 1:** Genetic variants among 88 alleles

Genetic variants distribution among 88 alleles					
	Genotype *null*	Genotype A	Genotype B	Genotype D	*p*
	*n* = 12	*n* = 42	*n* = 22	*n* = 12	
del	9 (75.0)	10 (23.8)	8 (36.4)	2 (16.7)	< 0.001
I172N	0	0	12 (54.5)	0	< 0.001
I2G	0	30 (71.4)	2 (9.1)	2 (16.7)	< 0.001
STOP codon	3 (25.0)	0	0	0	< 0.001
other	0	2 (4.8)	0	8 (66.7)	< 0.001

Categorical data were compared with the chi-square test

### Genotype-phenotype correlations

All genotypes were classified into four groups, as follows: *null*, group A, group B, and group D. Six patients were assigned to group *null*, 21 patients presented genotype A, and 11 had genotype B, while six patients were classified as genotype D.

Patients assigned to groups *null* and A were assumed to present a SW phenotype. Patients in genotype group B (with suspected sufficient residual 21-hydroxylase activity) were predicted to have the SV phenotype. Patients in group C were hypothesized to have the NCCAH clinical manifestation (but they were excluded from the study). Severe genotypes (*null* and A) demonstrated a good correlation with the expected phenotype, with positive predictive value (PPV) of 100 and 90.5%, respectively, whereas the less severe genotype B demonstrated a lower correlation (with PPV 66.7%). Nine patients presented a different phenotype from what had been expected (seven in the SW group and two in SV).

There were no differences in anthropometric data among the groups. In our study, patients with genotypes *null*, A, and B were overweight, with a BMI of 27.2 ± 8.0, 27.3 ± 5.9, and 28.3 ± 6.8 kg/m2, respectively. Age at menarche was also comparable. Blood glucose values were higher in group A, with a trend towards statistical significance (*P*-value 0.056). The clinical and molecular data according to genotypes are illustrated in Table 2.

**Table 2:** Clinical and molecular data according to genotypes

	Genotype *null*	Genotype A	Genotype B	Genotype D	*p*
	*n* = 6	*n* = 21	*n* = 11	*n* = 6	
Male sex (N [%])	2 (33.3)	7 (33.3)	3 (36.4)	1 (16.7)	0.861
CCAH type (N [%]):					0.010
SV	0 (0.0)	2 (9.5)	4 (36.4)	4 (66.7)	
SW	6 (100.0)	19 (90.5)	7 (63.6)	2 (33.3)	
Age of inclusion in the study (years)(Me [Q1; Q3])	20.5(19.0; 26.0)	28.0 (20.0; 31.0)	23.0 (20.0; 48.0)	24.0 (20.0; 28.0)	0.756
Menarche (years) (Me [Q1; Q3])	13.5 (12.5; 17.0)	13.0 (12.0; 14.5)	12.5 (11.0; 14.0)	12.0 (11.0; 14.0)	0.582
Height (cm)($\overline{x}$ ± SD)	161.3 ± 11.7	159.4 ± 11.9	161.6 ± 4.9	160.2 ± 10.2	0.978
BMI (kg/m2) ($\overline{x}$ ± SD)	27.2 ± 8.0	27.3 ± 5.9	28.3 ± 6.8	22.0 ± 3.0	0.187
17-OHP serum concentration (nmol/L) (Me [Q1; Q3])	24.5 (6.0; 43.9)	28.7 (8.8; 60.5)	18.8 (14.2; 60.5)	14.2 (4.8; 33.0)	0.874
Fasting glucose (mmol/l) ( ± SD)	4.5 ± 0.2	4.8 ± 0.5	4.4 ± 0.6	4.4 ± 0.4	0.056
Expected effect(N [%]):					0.164
Full blown	6 (100.0)	18 (85.7)	11 (100.0)	4 (66.7)	
Carrier	0 (0.0)	3 (14.3)	0 (0.0)	2 (33.3)	
Maximal residual enzyme activity:					< 0.001
0%	6 (100)	1 (4.8)	0	0	
< 1%	0	18 (85.7)	0	0	
1%	0	2 (9.5)	0	0	
2–11%	0	0	11 (100)	0	
Unknown	0	0	0	6 (100)	
Treatment					
Hydrocortisone (N (%])	6 (100.0)	19 (90.5)	10 (90.9)	5 (83.3)	0.802
Dexamethasone (N [%])	6 (100.0)	15 (71.4)	7 (63.6)	2 (33.3)	0.101
Prednisone (N [%])	0 (0.0)	7 (33.3)	0 (0.0)	0 (0.0)	0.031
Fludrocortisone (N [%])	6 (100.0)	17 (81.0)	8 (72.7)	3 (50.0)	0.213

*CCAH*, classical congenital adrenal hyperplasia; *SV*, simple virilizing; *SW*, salt-wasting; *17-OHP*, 17-hydroxyprogesterone. Continuous data were compared using the Kruskal-Wallis. Categorical data were compared with the chi-square test

### Novel variant

One novel variant was found in one allele in the study group, namely, c.[878G>T] (p.Gly293Val). This variant has not yet been described in the literature. However, another variant at this position, c.878G>A (p.Gly293Asp), has been associated with CCAH and shown to result in residual enzyme activity of < 1% [17].

We used bioinformatics tools to predict the effect of the variant detected in our study on protein function. The identified variant, c.[878G>T], has been predicted to be deleterious by PROVEAN (scored −8.38 at a cutoff of −2.5) [18] and damaging by SIFT (scored 0.000 at a cutoff of 0.05) [19]. Also according to the Bayes classifier applied in MutationTaster, the identified variant has been predicted to be disease-causing [20].

The clinical severity of the new variant could be deduced from the patient’s phenotype since he had a severe alteration (deletion of most of the gene) of the other allele.

## Discussion

This study identifies the spectrum and frequencies of *CYP21A2* variants as well as genotype-phenotype correlations in a group of 48 adult patients with CCAH due to 21-hydroxylase deficiency treated in the Department of Endocrinology at the University Hospital in Krakow, Poland. To the best of our knowledge, our study is the first published report on the spectrum and frequency of *CYP21A2* genetic alterations in the Polish population. As the distribution of *CYP21A2* variants differs between individual populations, the results of the study may be a valuable tool in genetic counseling not only in Polish patients with CCAH but also in populations of the entire European area.

The most common genetic variant in the studied group was I2G, followed by whole or partial gene copy deletion. In a study of 155 CAH patients from southern Germany (92 SW and 52 SV), I2G was also mentioned as the most common genetic variant [13]. A high frequency of this alteration was also observed by authors from Croatia [21], Turkey [22], India [23], Cuba [24], and China [25, 26]. In previous studies from Latin American countries (Argentina [27], Brazil [28]), and Portugal [29]), p.Val282Leu, which is the most frequent variant in NCCAH, was defined as the most common genetic alteration. Because only CCAH patients were enrolled in the present study, the latter variant accounted for only 3.41% of cases. It is believed that these differences in genotype frequencies in different countries may result from the heterogeneity/homogeneity of the studied population and different proportions of particular types of CAH in published series. The most common genetic alterations reported in several previous studies are summed up in Table 3.

**Table 3:** Most common genetic alterations reported in several previous studies

Country	Study (first author)	Year of publication	Centers included in the study	Total CAH cases (CCAH and NCAH)	CCAH		Most common genetic alteration	Frequency (%)
					SW	SV		
Germany	Riedl S.	2019	Multicenter	538	386	98	Del/conversions	29.6
Austria								
Germany	Krone N.	2000	Multicenter	155	92	52	I2G	30.3
Croatia	Dumic KK.	2017	Multicenter	93	66	23	I2G	35.5
Turkey	Turan I.	2020	Single center	113	82	24	I2G	38.4
India	Khajuria R.	2017	Single center	55	19	30	I2G	20.0
China	Xu C.	2019	Single	72	47	11	I2G	33.0
	Wang R.	2016	center	230	142	60	I2G	35.0
			Single center					
Serbia	Milacic I.	2015	Multicenter	61	19	15	I2G	18.5
Cuba	Espinosa Reyes TM.	2020	Single center	31	16	12	I2G	38.7
Argentina	Marino R.	2011	Multicenter	454	162	75	p.Val282Leu	26.2
Brazil	De Carvalho DF.	2016	Single center	480	158	116	p.Val282Leu	26.6
Portugal	Santos-Silva R.	2019	Multicenter	212	61	24	p.Val282Leu	41.3
Cyprus	Neocleous V.	2019	Multicenter	120	11	7	p.Val282Leu	60.0
USA	New MI.	2013	Multicenter	1500	606	187	p.Val282Leu	23.9

In our study group, a good correlation between genotype and phenotype was observed in group *null* (patients with alterations in both alleles resulting in 0% residual enzyme activity) and group A (0–1% residual enzyme function). Only in seven patients with SW and in two with SV was there no concordance between genotype and phenotype. Genotype-phenotype correlations measured by PPV were as follows: 100% in group *null*, 90.5% in group A, and 66.7% in group B. Results of previous studies confirm a good correlation between the genotype and the observed CAH phenotype [26, 28, 30–33]. Previous studies reported 100% concordance in *null*, 80–96% in A, and about 50–87% in B genotypes [13, 27, 34, 35]. In a huge cohort study by New at al., based on 1507 families with CAH, a genotype-phenotype non-concordance was observed in 50% of cases [36]. A recent German-Austrian study, which enrolled the largest European cohort of CAH patients from 28 centers (538 CAH cases), has reported a poor correlation in the less severe genotypes B (46%) and C (58%) [37].

In four patients of our study, extensive rearrangements were detected, and numerous pathogenic variants were found. These patients have been excluded from further analysis since it was impossible to determine the allelic distributions of these variants without performing an evaluation of the patients’ parents, who were unavailable for genetic testing. In the latter group, one novel variant has been identified.

In our study group, there were more female than male patients. This is in agreement with previous data, according to which a substantial proportion of male patients remain undiagnosed [36]. In our study, adult patients were included in whom the diagnosis of CCAH was made years ago when neonatal screening was not available in Poland. This higher proportion of women is typical for countries where neonatal screening has not been routinely used. We believe that this trend may be reversed in future given that neonatal screening for CAH was introduced in Poland in 2016 [38].

In all groups *null*, A, B, and D, the patients’ BMI was > 25 kg/m2. The tendency for obesity in CCAH accords with the data reported in previous studies [39, 40]. Fasting blood glucose concentration was higher in group A, with a trend towards statistical significance. One of the largest studies on cardiometabolic complications also demonstrated higher frequencies of obesity and diabetes (mainly type 2) in CAH patients [41].

One novel variant was found in one allele in the study group, namely, c.[878G>T] (p.Gly293Val). By means of bioinformatics tools, the identified variant has been predicted to be pathogenic.

The patient had a severe alteration (deletion of most of the gene) on another allele. The clinical severity of the new variant could be deduced from the patient’s phenotype; the diagnosis of SW in this patient was established in the neonatal period, when he presented symptoms of adrenal crisis and required glucocorticoid and mineralocorticoid therapy.

However, the small study group being a limitation of this study, the observed non-concordance between genotype and phenotype requires further investigation.

Due to the fact that neonatal screening has been widely introduced in most countries, the number of patients diagnosed with CCAH is expected to increase in the future. The results of the present study, and particularly the description of the novel variant, may contribute to a better understanding of the disease. Moreover, the presented data can be useful for the prediction of phenotype based on genotype and may be helpful not only in genetic counseling but also in making treatment decisions. The practical implication of the data is that special attention must be paid to patients with no or very low 21-hydroxylase activity determined by genotype, in whom in cases of insufficient doses of corticoids or concomitant acute diseases adrenal crisis may occur.

## Conclusions

The majority of cases in our study were characterized by a strong genotype-phenotype correlation. Variant frequencies of affected alleles were similar to those of data previously reported for other countries of the region. The most common genetic variant in the study cohort was I2G, followed by large deletions and I172N. One novel variant, c.[878G>T] (p.Gly293Val), has been identified and characterized by means of bioinformatics tools.

## References

[1] Merke DP, Auchus RJ (2020). Congenital adrenal hyperplasia due to 21-hydroxylase deficiency. N Engl J Med.

[2] Speiser PW, Arlt W, Auchus RJ, Baskin LS, Conway GS, Merke DP, Meyer-Bahlburg HFL, Miller WL, Murad MH, Oberfield SE, White PC (2018). Congenital adrenal hyperplasia due to steroid 21-hydroxylase deficiency: an endocrine society clinical practice guideline. J Clin Endocrinol Metab.

[3] El-Maouche D, Arlt W, Merke DP (2017). Congenital adrenal hyperplasia. Lancet.

[4] Krone N, Rose IT, Willis DS, Hodson J, Wild SH, Doherty EJ, Hahner S, Parajes S, Stimson RH, Han TS, Carroll PV, Conway GS, Walker BR, MacDonald F, Ross RJ, Arlt W (2013). Genotype-phenotype correlation in 153 adult patients with congenital adrenal hyperplasia due to 21-hydroxylase deficiency: analysis of the United Kingdom congenital adrenal hyperplasia adult study executive (CaHASE) cohort. J Clin Endocrinol Metab.

[5] Trakakis E, Basios G, Trompoukis P, Labos G, Grammatikakis I, Kassanos D (2010). An update to 21-hydroxylase deficient congenital adrenal hyperplasia. Gynecol Endocrinol.

[6] Pang S, Murphey W, Levine LS, Spence DA, Leon A, LaFranchi S, Surve AS, New MI (1982). A pilot newborn screening for congenital adrenal hyperplasia in Alaska. J Clin Endocrinol Metab.

[7] Speiser PW, White PC (2003). Congenital adrenal hyperplasia. N Engl J Med.

[8] Speiser PW, Chawla R, Chen M, Diaz-Thomas A, Finlayson C, Rutter MM, Sandberg DE, Shimy K, Talib R, Cerise J, Vilain E, Délot EC (2020). Newborn screening protocols and positive predictive value for congenital adrenal hyperplasia vary across the United States. Int J Neonatal Screen.

[9] White PC, New MI, Dupont B (1986). Structure of human steroid 21-hydroxylase genes. Proc Natl Acad Sci U S A.

[10] Parsa AA, New MI (2017). Steroid 21-hydroxylase deficiency in congenital adrenal hyperplasia. J Steroid Biochem Mol Biol.

[11] Concolino P, Costella A (2018). Congenital adrenal hyperplasia ( CAH ) due to 21-hydroxylase deficiency: a comprehensive focus on 233 pathogenic variants of CYP21A2 gene. Mol Diagn Ther.

[12] Simonetti L, Bruque C, Fernández C, Benavides-Mori B, Delea M, Kolomenski JE, Espeche LD, Buzzalino ND, Nadra AD, Dain L (2018). CYP21A2 mutation update: comprehensive analysis of databases and published genetic variants. Hum Mutat.

[13] Krone N, Braun A, Roscher AA, Knorr D, Schwarz HP (2000). Predicting phenotype in steroid 21-hydroxylase deficiency. Comprehensive genotyping in 155 unrelated, well defined patients from southern Germany. Clin Endocrinol Metab.

[14] Xu Z, Chen W, Merke DP, McDonnell NB (2013). Comprehensive mutation analysis of the CYP21A2 gene: an efficient multistep approach to the molecular diagnosis of congenital adrenal hyperplasia. J Mol Diagn.

[15] Wedell A, Thilén A, Ritzén EM, Ritzén EM, Stengler B, Luthman H (1994). Mutational spectrum of the steroid 21-hydroxylase gene in Sweden: implications for genetic diagnosis and association with disease manifestation. J Clin Endocrinol Metab.

[16] Speiser PW, Dupont J, Zhu D, Serrat J, Buegeleisen M, Tusie-Luna MT, Lesser M, New MI, White PC (1992). Disease expression and molecular genotype in congenital adrenal hyperplasia due to 21-hydroxylase deficiency. J Clin Invest.

[17] Tardy V, Menassa R, Sulmont V, Lienhardt-Roussie A, Lecointre C, Brauner R, David M, Morel Y (2010). Phenotype-genotype correlations of 13 rare CYP21A2 mutations detected in 46 patients affected with 21-hydroxylase deficiency and in one carrier. J Clin Endocrinol Metab.

[18] Choi Y, Sims GE, Murphy S, Miller JR, Chan AP (2012). Predicting the functional effect of amino acid substitutions and indels. PLoS One.

[19] Kumar P, Henikoff S, Ng PC (2009). Predicting the effects of coding non-synonymous variants on protein function using the SIFT algorithm. Nat Protoc.

[20] Schwarz JM, Cooper DN, Schuelke M, Seelow D (2014). Mutationtaster2: mutation prediction for the deep-sequencing age. Nat Methods.

[21] Dumic KK, Grubic Z, Yuen T, Wilson RC, Kusec V, Barisic I, Stingl K, Sansovic I, Skrabic V, Dumic M, New MI (2017). Molecular genetic analysis in 93 patients and 193 family members with classical congenital adrenal hyperplasia due to 21-hydroxylase deficiency in Croatia. J Steroid Biochem Mol Biol.

[22] Turan I, Tastan M, Boga DD, Gurbuz F, Kotan LD, Tuli A, Yüksel B (2020). 21-hydroxylase deficiency: mutational spectrum and genotype – phenotype relations analyses by next-generation sequencing and multiplex ligation- dependent probe amplification. Eur J Med Genet.

[23] Khajuria R, Walia R, Bhansali A, Prasad R (2017). The spectrum of CYP21A2 mutations in congenital adrenal hyperplasia in an Indian cohort. Clin Chim Acta.

[24] Espinosa Reyes TM, Collazo Mesa T, Lantigua Cruz PA, Agramonte Machado A, Domínguez Alonso E, Falhammar H (2020). Molecular diagnosis of patients with congenital adrenal hyperplasia due to 21-hydroxylase deficiency. BMC Endocr Disord.

[25] Xu C, Jia W, Cheng X, Ying H, Chen J, Xu J, Guan Q, Zhou X, Zheng D, Li G, Zhao J (2019). Genotype – phenotype correlation study and mutational and hormonal analysis in a Chinese cohort with 21-hydroxylase deficiency. Mol Genet Genomic Med.

[26] Wang R, Yu Y, Ye J, Han L, Qiu W, Zhang H, Liang L, Gong Z, Wang L, Gu X (2016). 21-hydroxylase deficiency-induced congenital adrenal hyperplasia in 230 Chinese patients: genotype-phenotype correlation and identification of nine novel mutations. Steroids.

[27] Marino R, Ramirez P, Galeano J, Perez Garrido N, Rocco C, Ciaccio M, Warman DM, Guercio G, Chaler E, Maceiras M, Bergadá I, Gryngarten M, Balbi V, Pardes E, Rivarola MA, Belgorosky A (2011). Steroid 21-hydroxylase gene mutational spectrum in 454 Argentinean patients : genotype – phenotype correlation in a large cohort of patients with congenital adrenal hyperplasia. Clin Endocrinol.

[28] de Carvalho DF, Miranda MC, Gomes LG, Madureira G, Marcondes JA, Billerbeck AE, Rodrigues AS, Presti PF, Kuperman H, Damiani D, Mendonca BB, Bachega TA (2016). Molecular CYP21A2 diagnosis in 480 Brazilian patients with congenital adrenal hyperplasia before newborn screening introduction. Eur J Endocrinol.

[29] Santos-Silva R, Cardoso R, Lopes L, Fonseca M, Espada F, Sampaio L, Brandão C, Antunes A, Bragança G, Coelho R, Bernardo T, Vieira P, Morais R, Leite AL, Ribeiro L, Carvalho B, Grangeia A, Oliveira R, Oliveira MJ, Rey V, Rosmaninho-Salgado J, Marques B, Garcia AM, Meireles A, Carvalho J, Sequeira A, Mirante A, Borges T (2019). CYP21A2 gene pathogenic variants: a multicenter study on genotype – phenotype correlation from a Portuguese pediatric cohort. Horm Res Paediatr.

[30] Narasimhan ML, Khattab A (2019). Genetics of congenital adrenal hyperplasia and genotype-phenotype correlation. Fertil Steril.

[31] Milacic I, Barac M, Milenkovic T, Ugrin M, Klaassen K, Skakic A, Jesic M, Joksic I, Mitrovic K, Todorovic S, Vujovic S, Pavlovic S, Stojiljkovic M (2015). Molecular genetic study of congenital adrenal hyperplasia in Serbia: novel p.Leu129Pro and p.Ser165Pro CYP21A2 gene mutations. J Endocrinol Investig.

[32] Zhang B, Lu L, Lu Z (2017). Molecular diagnosis of Chinese patients with 21-hydroxylase deficiency and analysis of genotype–phenotype correlations. J Int Med Res.

[33] Neocleous V, Fanis P, Toumba M, Stylianou C, Picolos M, Andreou E, Kyriakou A, Iasonides M, Nicolaou S, Kyriakides TC, Tanteles GA, Skordis N, Phylactou LA (2019). The spectrum of genetic defects in congenital adrenal hyperplasia in the population of Cyprus: a retrospective analysis. Horm Metab Res.

[34] Dundar A, Bayramov R, Onal MG, Akkus M, Dogan ME, Kenanoglu S, Cerrah Gunes M, Kazimli U, Ozbek MN, Ercan O, Yildirim R, Celmeli G, Parlak M, Dundar I, Hatipoglu N, Unluhizarci K, Akalin H, Ozkul Y, Saatci C, Dundar M (2019). The molecular basis and genotype–phenotype correlations of congenital adrenal hyperplasia (CAH) in Anatolian population. Mol Biol Rep.

[35] Finkielstain GP, Chen W, Mehta SP, Fujimura FK, Hanna RM, Van Ryzin C, McDonnell NB, Merke DP (2011). Comprehensive genetic analysis of 182 unrelated families with congenital adrenal hyperplasia due to 21-hydroxylase deficiency. J Clin Endocrinol Metab.

[36] New MI, Abraham M, Gonzalez B, Dumic M, Razzaghy-Azar M, Chitayat D, Sun L, Zaidi M, Wilson RC, Yuen T (2013). Genotype-phenotype correlation in 1,507 families with congenital adrenal hyperplasia owing to 21-hydroxylase deficiency. Proc Natl Acad Sci U S A.

[37] Riedl S, Röhl FW, Bonfig W, Brämswig J, Richter-Unruh A, Fricke-Otto S, Bettendorf M, Riepe F, Kriegshäuser G, Schönau E, Even G, Hauffa B, Dörr HG, Holl RW, Mohnike K, AQUAPE CAH Study Group (2019). Genotype/phenotype correlations in 538 congenital adrenal hyperplasia patients from Germany and Austria: discordances in milder genotypes and in screened versus prescreening patients. Endocr Connect.

[38] Ginalska-Malinowska M (2018). Classic congenital adrenal hyperplasia due to 21-hydroxylase deficiency - the next disease included in the neonatal screening program in Poland. Dev Period Med.

[39] Gomes LG, Mendonca BB, Bachega TASS (2020). Long-term cardio-metabolic outcomes in patients with classical congenital adrenal hyperplasia: is the risk real?. Curr Opin Endocrinol Diabetes Obes.

[40] Paizoni L, Auer MK, Schmidt H, Hübner A, Bidlingmaier M, Reisch N (2020). Effect of androgen excess and glucocorticoid exposure on metabolic risk profiles in patients with congenital adrenal hyperplasia due to 21-hydroxylase deficiency. J Steroid Biochem Mol Biol.

[41] Falhammar H, Frisén L, Hirschberg AL, Norrby C, Almqvist C, Nordenskjöld A, Nordenström A (2015). Increased cardiovascular and metabolic morbidity in patients with 21-hydroxylase deficiency: a Swedish Population-Based National Cohort Study. J Clin Endocrinol Metab.

